# Id1 enhances human ovarian cancer endothelial progenitor cell angiogenesis via PI3K/Akt and NF-κB/MMP-2 signaling pathways

**DOI:** 10.1186/1479-5876-11-132

**Published:** 2013-05-29

**Authors:** Yajuan Su, Lingjuan Gao, Lichen Teng, Ying Wang, Jialin Cui, Shiyun Peng, Songbin Fu

**Affiliations:** 1 Department of clinical laboratory, the affiliated tumor hospital, Harbin Medical University, Harbin, China; 2 Department of clinical laboratory, Nanjing Maternity and Child Health Care hospital, Nanjing Medical University, Nanjing, China; 3 Laboratory of Medical Genetics, Harbin Medical University, Harbin, 150081, China

**Keywords:** Id1, Endothelial progenitor cells, Angiogenesis, PI3K/Akt, NF-κB/MMP-2

## Abstract

**Background:**

Endothelial progenitor cells (EPCs) contribute to tumor angiogenesis and growth. We previously reported that over-expression of an inhibitor of DNA binding/differentiation 1 (Id1) in EPCs can enhance EPC proliferation, migration, and adhesion. In this study, we investigated the role of Id1 in EPC angiogenesis in patients with ovarian cancer and the underlying signaling pathway.

**Methods:**

Circulating EPCs from 22 patients with ovarian cancer and 15 healthy control subjects were cultured. Id1 and matrix metalloproteinase-2 (MMP-2) expression were analyzed by real-time reverse transcription-polymerase chain reaction (RT-PCR) and western blot. EPC angiogenesis was detected by tube formation assays. Double-stranded DNA containing the interference sequences was synthesized according to the structure of a pGCSIL-GFP viral vector and then inserted into a linearized vector. Positive clones were identified as lentiviral vectors that expressed human Id1 short hairpin RNA (shRNA).

**Results:**

Id1 and MMP-2 expression were increased in EPCs freshly isolated from ovarian cancer patients compared to those obtained from healthy subjects. shRNA-mediated Id1 down-regulation substantially reduced EPC angiogenesis and MMP-2 expression. Importantly, transfection of EPCs with Id1 in vitro induced phosphorylation of Akt (p-Akt) via phosphoinositide 3-kinase and increased the expression of MMP-2 via NF-κB. Blockage of both pathways by specific inhibitors (LY294002 and PDTC, respectively) abrogated Id1-enhanced EPC angiogenesis.

**Conclusions:**

Id1 can enhance EPC angiogenesis in ovarian cancer, which is mainly mediated by the PI3K/Akt and NF-κB/MMP-2 signaling pathways. Id1 and its downstream effectors are potential targets for treatment of ovarian cancer because of their contribution to angiogenesis.

## Background

Tumor angiogenesis is recognized as a critical step in tumor progression through which an initially small, localized or non-invasive tumor gradually develops into a large, invasive, metastatic one. Previous studies have shown that bone marrow (BM)-derived EPCs participate in tumor angiogenesis, which accelerates tumor growth [1-3]. Furthermore, EPCs control the angiogenic switch in mouse lung metastasis [4]. Currently, the reasons for ovarian cancer EPC angiogenesis are poorly understood.

Inhibitors of differentiation 1 (Id1) belong to the helix loop helix (HLH) transcription factors family. Maw et al. [5] showed that the level of Id1 expression was positively related to the degree of malignancy in ovarian cancer. A study by Lyden et al. [6] confirmed that Id1 and Id3 played an important role in the vascular endothelial growth factor (VEGF) signal pathway, which is related to angiogenesis. In Id1 knock-out mice, it appeared that tumor growth was significantly inhibited due to an angiogenesis defect. BM-derived EPCs participated in the formation of new blood vessels [7], suggesting that EPCs have a close relationship with Id1. A recent report showed that tumor could induce high expression of Id1 in EPCs derived from BM but not in other cells, suggesting that Id1 might be a key factor for EPCs. A defect of Id1 in BM could lead to decreased numbers of EPCs in peripheral blood, block tumor angiogenesis, and further suppress tumor development [8]. Thus, Id1 may mediate angiogenesis of EPCs however, the mechanism is still poorly understood.

In a previous study, we used real-time RT-PCR to examine mRNA expression of Id1 in EPCs of 25 patients with ovarian cancer [9]. Western blot analysis revealed a higher Id1 expression in human ovarian cancer EPCs than in cells from 20 healthy controls. Compared to healthy controls, ovarian cancer patients showed increased migration and adhesion of EPCs. Statistical analyses revealed that ovarian cancer enhanced proliferation, migration, and adhesion of EPCs [9,10].

In the present study, we examined whether the over-expression of Id1 can enhance angiogenesis in cultured human ovarian cancer EPCs. We hypothesized that Id1 is linked to the angiogenesis of ovarian cancer EPCs via regulation of the NF-κB/matrix metalloproteinase-2 (MMP-2) and PI3K/Akt pathways. Our in vitro data showed that Id1 up-regulated MMP-2 via a NF-κB–dependent mechanism and simultaneously activated the Akt pathway via PI3K, contributing to EPC angiogenesis. These findings show the existence of an Id1/NF-κB/MMP-2/Akt signaling axis in ovarian cancer EPC angiogenesis.

## Methods

### Patients

This study was approved by the local ethics committee in China and informed consent was obtained from all study participants. Twenty-two patients (median age, 46 years age range, 20–62 years) with histologically proven ovarian cancer, including serous cancer (n=14), mucinous cancer (n = 5), and endometrioid cancer (n = 3), were studied along with a control group of 15 healthy women (age range, 18–34 years). Patients who were diagnosed with ovarian cancer had no additional malignant, inflammatory, or ischemic disease; wounds; or ulcers that could influence the number of EPCs.

### Cell culture

The Ethics Committee of the Harbin Medical University approved the study protocol. EPC culture and identification were described in our previous paper [9]. Total mononuclear cells (MNCs) were isolated from 20 ml samples of human peripheral blood from patients with ovarian cancer and healthy women by density gradient centrifugation with Histopaque-1077 (density 1.077 g/ml; Sigma). MNCs were plated in 1 ml endothelial growth medium (EGM-2; Lonza) on fibronectin-coated (Sigma) 24-well plates. After 24 h of culturing, unattached cells were discarded and attached cells were cultured as before. Medium was replaced every 2 days thereafter, and each colony/cluster was followed up. After 7 days in culture, colony forming cells were recognized as attached spindle-shaped cells. The adherent cells were incubated with DiI-acLDL (Molecule Probes) and then fixed in 2% paraformaldehyde and counterstained with fluorescein isothiocyanate (FITC)-labeled lectin from *Ulex europaeus* agglutinin (UEA-1) (Sigma). The fluorescent images were recorded under a fluorescent microscope. Cells also were characterized by immunofluorescence staining for von Willebrand factor (vWF) and expression of CD31 and vascular endothelial growth factor receptor-2 (VEGFR2) (Becton Dickinson).

Human umbilical vein endothelial cells (HUVECs) (purchased from Cambrex Bio Science, Walkersville, MD) were cultured in medium 199 containing 10% FBS, penicillin (100 U/ml), streptomycin (100 mg/ml), heparin (50 mg/ml), and endothelial cell growth supplement (50 mg/ml). Third to seventh passages of HUVECs were used for experiments. HUVECs were maintained in a 5% CO_2_ incubator at 37°C.

### Quantitative real-time RT-PCR

Total RNA isolation and cDNA synthesis from cultured EPCs were performed using Trizol and the SuperScript II Reverse Transcriptase kit (Invitrogen, USA) according to the manufacturer’s instructions. Real-time PCR was performed with the Mx3000p Real Time PCR System (Stratagene, USA) using the following thermal cycling conditions: 10 sec at 95°C followed by 40 cycles of 15 sec at 95°C, 20 sec at 60°C, and 7 sec at 72°C. SYBR® GreenER qPCR SuperMix Universal S (Invitrogen, USA) (25 μl) were performed in triplicate. A no-template control (replacing RNA with water) was used as a negative control. Id1, MMP-2 and MMP-9 mRNA in the EPCs was determined by relative quantitation, interpolating from a standard curve of template DNA of known concentration and then normalized using β-actin as an internal control. Data were analyzed by 2^-ΔΔCt^.

The primer sequences used for real-time PCR were as follows:

Id1,5-GTAAACGTGCTGCTCTACGACATGA-3 and 5-AGCTCCAACTGAAGGTCCCTGA-3; mmp-2, 5-TTGACAACAACGGTACTGCTAC-3 and 5-TGGTGAACACTGTGCTGATTAC-3; mmp-9, 5-ATCACTACTACCGCATTACCAC-3 and 5-TCACGAATATAGTGGCGATATC-3; β-actin, 5-TGGCACCCAGCACAATGAA-3 and 5-CTAAGTCATAGTCCGCCTAGAAGCA-3.

### Western blots

The EPCs were collected with sample buffer. Cell lysates were centrifuged at 10000 g for 10 min at 4°C and the supernatant was stored at −70°C. Protein concentrations were determined with a Bio-Rad kit. 50-μg aliquots of protein were subjected to 12% and 6% SDS-PAGE gels. Then the protein was blotted onto a polyvinylidene fluoride (PVDF) membrane. Primary antibodies against Id1 (1:1000, Becton Dickinson), MMP-2 (1:1000, Becton Dickinson), MMP-9 (1:1000, Becton Dickinson), Phospho-65 (1:1000, Cell Signaling), Phospho-Akt (ser473) (1:1000, Cell Signaling), Total-Akt (1:2000, Cell Signaling), and β-actin (1:2000, Becton Dickinson) were used according to the manufacturer’s recommendations. After washing the membrane, a second antibody (HRP-conjugated anti-mouse IgG) (1:2000, Becton Dickinson) was used to detect Id1, mmp-2, mmp-9, p-65, Phospho-Akt, Total-Akt, and β-actin. The bands were visualized using Pierce ECL Western Blotting Substrate (Thermo Fisher Scientific Inc., Rockford, IL, USA) with 5 to 30 min exposure after washing the membrane. β-actin was used as the protein loading control.

### Molecular reagents

The Id1 cDNA from an ovarian cancer specimen was cloned into a plasmid with enhanced green fluorescent protein (GFP) (Clontech), and lentiviral vector expressing Id1-specific short hairpin RNA (shRNA) were constructed as described previously (9). Pyrrolidine dithiocarbamate (PDTC) was used as an alternative inhibitor of the NF-κB activity. LY294002 was used as a specific PI3K inhibitor.

### In vitro transduction of EPCs

For lentiviral transduction, the primary EPCs were passaged into 6-well plates at a density of 1 × 10^5^ cells/well. When cells reached 30% confluence (typically on the third day after subculturing), the medium was replaced with 1 ml of fresh medium containing lentivirus at an MOI of 150 and 6 μg/ml polybrene (Gikai gene company, Shanghai, China). The medium was replaced with fresh medium on the following day. Five days after transduction, cells were analyzed by flow cytometry using a BD FACSCalibur™ cell analyzer (BD Biosciences). The percentage of GFP-positive cells and mean fluorescence intensity (MFI) of GFP-positive cells were determined with WinMDI 2.8 software (J. Trotter, Flow Cytometry Core Facility, Scripps Research Institute, La Jolla, CA). Means and standard deviations from experiments performed in triplicate are given.

### In vitro tube formation

In vitro tube formation assay was performed using the Matrigel basement membrane matrix (BD Biosciences). 1 ml/well Matrigel, kept on ice, was placed in 4-well culture plates. The plates were then incubated at 37°C for 30 min to allow Matrigel to solidify. About 2×10^4^ FITC-UEA-1-labeled EPCs were co-cultured with 4×10^4^ HUVECs on the preplated Matrigel. The number of FITC-UEA-1 EPCs incorporated to the tube was determined in five random high-power fields in duplicates. A tube was defined as a structure exhibiting a length four times its width.

### Luciferase assays

Cells cultured in a 12-well plate with 60% confluence were transfected with the Id1 cDNA at 1.4 μg/ml and co-transfected with NF-κB luciferase/β-galactosidase reporters, at 1.4 μg/ml for 16 h in the transfection medium and recovered in culture medium for 24 h. Cells were harvested for luciferase assays, as previously described [11]. The activity of NF-κB luciferase over β-galactosidase (internal control) is presented as a relative luciferase activity. The Tropix dual reporter kit (Applied Biosystems) was used with a Berthold TriStar flash injection luminometer.

### Statistical analysis

Statistical analyses were performed with Statistical Package for Social Sciences 13.0 software program (SPSS, USA). The Mann–Whitney U test and Student’s t-test were used to compare variables between the two groups. Multiple comparisons were analyzed by Anova followed by post-hoc analysis to adjust the significance level. Data are shown as means ± S.E. Statistical significance was considered as P < 0.05.

## Results

### Characterization of EPCs

**Figure 1:**
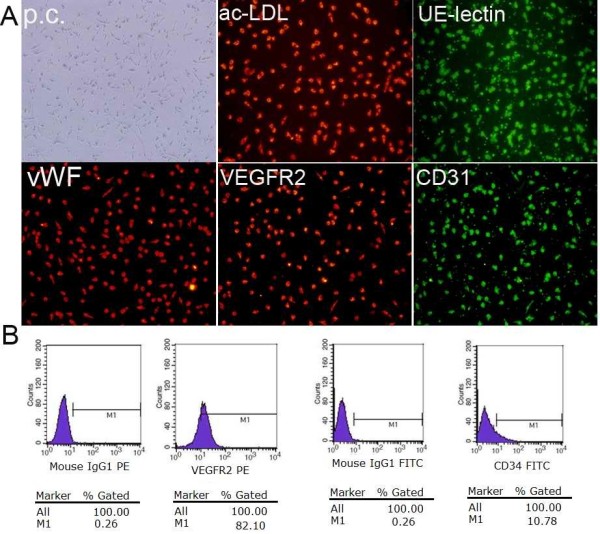
**Phenotypic characterization of EPCs from human peripheral blood.** (**A**) After 1 week in culture, EPCs were stained with DIL-labeled ac-LDL, FITC-conjugated *Ulex europaeus* lectin. vWF, VEGFR2 and CD31 analysis were assessed by immunofluorescence (× 20). (**B**) Detection of cell markers on EPCs by flow cytometry, from left to right: isotype-negative control, the percentage of VEGFR2-positive cells, isotypenegative control and the percentage of CD34-positive cells.

After 7 days of culture, ex vivo expanded EPCs derived from peripheral blood of healthy human volunteers and patients with ovarian cancer exhibited spindle-shaped morphology. EPCs were characterized as adherent and double positive for Dil-Ac-LDL uptake and lectin binding based on their appearance under a fluorescent microscope. A total of 93.8 ± 4.5% of adherent cells showed uptake of Dil-Ac-LDL and lectin binding after 7 days of culture. The endothelial phenotype of these expanded EPCs was further characterized by the expression of endothelial markers such as vWF, CD31, and VEGFR2. Immunofluorescence showed that the cells were positive for vWF, CD31, and VEGFR2 (Figure 1A).

We measured special molecular markers on the cell surface by flow cytometry to identify EPCs. A specific molecular marker that can be used strictly to isolate EPCs from other cells is lacking. EPCs can express various markers at different stages during development. Moreover, surface markers seems to differ in EPCs originating from different sources, so there may not be a simple surface marker on EPCs. However, CD34 and VEGFR-2 are widely considered to be surface markers of EPCs. In this study, we examined the expression of CD34 and VEGFR-2 on adherent cells derived from mononuclear cells cultured for 7 days using flow cytometry. The results showed that CD34-positive cells accounted for 8.32±1.49%, whereas, VEGFR2 -positive cells accounted for 80.37±4.03% (Figure 1B). Thus, the EPCs isolated can be defined as early-stage EPCs, although the CD34 expression of cells was low, which can differentiate as endothelial cells.

### Id1 increases EPCs angiogenesis in vitro

**Figure 2:**
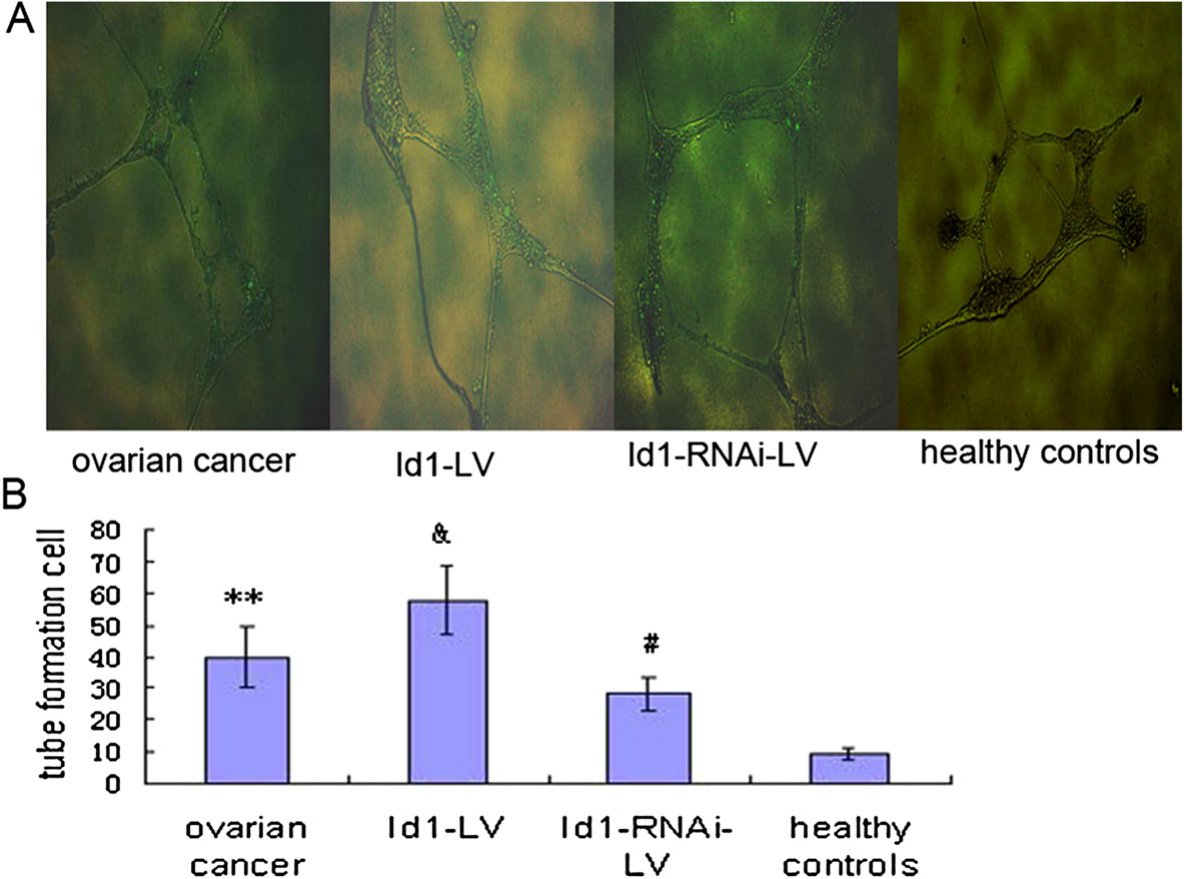
**Id1 increases EPC angiogenesis.** (**A**) Typical images of tube formation. (**B**) Id1 increases EPC angiogenesis. **p<0.01 vs. control, #, &p<0.05 vs. ovarian cancer.

EPC angiogenesis functions in ovarian cancer were examined by assessing tube formation. Tube formation in the Matrigel assay was markedly enhanced in EPCs. (Figure 2). We next examined whether over-expression of Id1 in EPCs can induce angiogenesis. Id1-LV and Id1-RNAi-LV were constructed, as previously reported by us (9). After the Id1-LV and Id1-RNAi-LV construct was transfected into EPCs, we performed the EPC tube formation analysis. Id1-LV and Id1-RNAi-LV were markedly increased and reduced EPC tube formation. EPC tube formation was significantly decreased by Id1 knock-down, compared to non-transfected control cells, as shown in Figure 2A-B. Taken together, these observations indicate that over-expression of Id1 can induce angiogenic processes in EPCs.

### PI3K/Akt and NF-kB are associated with Id1 and EPCs angiogenesis

**Figure 3:**
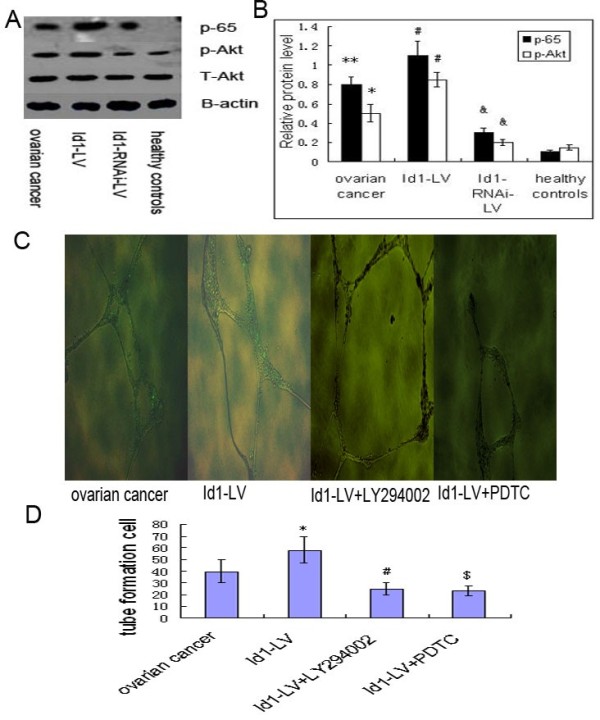
**Effects of LY294002 and PDTC on EPC angiogenesis functions.** (**A**) Typical western blot images showing protein expression of p-65,p-Akt and t-Akt (β-actin is shown as a housekeeping control). (**B**) The graph showing the relative p-65 and p-Akt protein levels normalized to actin. The results are expressed as the mean ± S.E. **p<0.01 , *p < 0.05 vs. control, #,&p < 0.05 vs. ovarian cancer. (**C**) Typical images of tube formation assay. (**D**) Accumulated data showing EPCs angiogenesis functions. * , #,$ p<0.05 vs. ovarian cancer.

EPCs use a broad spectrum of angiogenesis mechanisms to achieve enhanced tumor metastasis (8). To begin to determine which signaling transduction pathways might participate in Id1-mediated cell angiogenesis in EPCs, we investigated the PI3K/AKT pathway using pharmacological inhibitors. Elevated AKT-Ser473 phosphorylation was observed in EPCs, Id1-LV and Id1-RNAi-LV were markedly increased and reduced AKT-Ser473 phosphorylation in EPC (Figure 3A-B). EPCs that were transfected with Id1 were used in tube formation assay. EPCs were transfected with Id1 and then treated with PI3K inhibitor (LY294002 at 50 μmol/L) and evaluated. LY294002 significantly reduced EPC tube formation by Id1 (Figure 3C-D). These results indicate that Id1-induced EPC angiogenesis is mediated by the PI3K/AKT pathway. Because expression of phosphorylated 65 was elevated in EPCs (Figure 3A-B), we examined whether Id1 stimulation could activate NF-kB in EPCs. Cells were transfected with Id1 in the presence and absence of NF-κB inhibitors PDTC. PDTC abrogated the Id1-induced angiogenesis as judged by tube formation (Figure 3C-D). These data indicate that Id1increases p-Akt and activates NF-κB, which in turn increases EPC angiogenesis.

### Id1 up-regulates MMP-2 via NF-κB in EPCs

**Figure 4:**
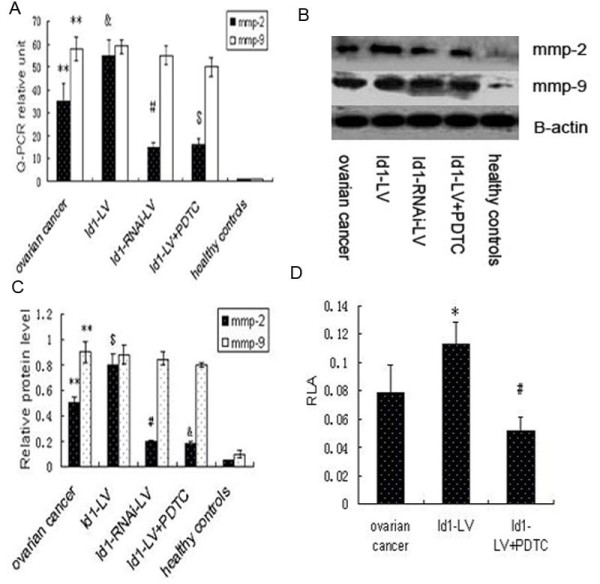
**Id1 regulates the expression of MMP-2 via NF-κB in EPCs.** (**A**) MMP-2 and MMP-9 mRNA expression by real-time RT-PCR. Data are expressed as means ± S.E. **p < 0.01 vs. control, #, &, $p < 0.05 vs. ovarian cancer. (**B**) Typical western blot images showing protein expression of MMP-2 and MMP-9 (β-actin is shown as a housekeeping control). (**C**) The graph showing the relative MMP-2 and MMP-9 protein levels normalized to β-actin. The results are expressed as the mean ± S.E. **p < 0.01 vs. control, #,&,$p < 0.05 vs. ovarian cancer. (**D**) Transfection of EPCs with Id1 significantly increased the promotor activity of NF-κB by luciferase assay. Increased promotor activity was abrogated by PDTC. RLA = relative luciferase activity. *p < 0.05 vs. ovarian cancer, #p < 0.05 vs. Id1-LV.

MMP-2 and MMP-9 are MMPs that are relevant to angiogenic processes. We examined MMP-2 and MMP-9 expression levels of EPCs. Basal expression levels of MMP-2 and MMP-9 mRNA and protein were significantly increased in EPCs (Figure 4A-D). After the Id1-LV and Id1-RNAi-LV construct was transfected into EPCs, we analyzed EPC MMP-2 and MMP-9 expression levels. Id1-LV and Id1-RNAi-LV, respectively, markedly increased and reduced EPC mRNA expression of MMP-2, but not MMP-9. Compared to non-transfected control cells, expression of MMP-2 was significantly decreased by Id1 knock-down, as shown in Figure 4A-C.

Because MMP-2 and Id1 were correlated with each other in EPCs, we postulated that Id1 might control the expression of MMP-2 in EPCs via NF-κB. To test this hypothesis, EPCs were transfected with Id1, co-transfected with NF-κB and β-galactosidase reporters, and harvested for evaluation of NF-κB promoter activity by luciferase assays and of MMP-2 by western blot. Id1 significantly increased NF-κB promoter activity, whereas PDTC abrogated Id1-induced NF-κB promoter activity (Figure 4D). Simultaneously, Id1 significantly increased the expression of MMP −2, and Id1-induced MMP −2 expression was abrogated by PDTC as shown by western lot (Figure 4B-C). This suggests that Id1 increases the expression of MMP-2 via NF-κB.

## Discussion

Angiogenesis is an important mechanism for tumorigenesis. Emerging evidence indicates that BM-derived EPCs participate in the tumor vascular network in different ways. They favor the formation of primitive tumor endothelium, control tumor growth, and promote the establishment of the pre-metastatic niche [12,13]. Moreover, the contribution of BM-derived EPCs to tumor neovascularization has been reported in mice and humans [14,15]. In our previous study, we found that EPCs from patients with ovarian cancer transfected with Id1-RNAi-LV displayed less proliferation, migration, and adhesion abilities compared to non-transfected control cells [9]. The proliferation, migration, and adhesion properties of ovarian cancer EPCs are attributable to the high expression of Id1, integrin α4 and p-Akt. Id1 contributes to this angiogenesis via the PI3K/Akt and integrin-α4 signaling pathways.

The molecular mechanism involved in EPC-induced tumor angiogenesis is poorly understood. VEGF and placental growth factor (PlGF) have been shown to contribute to EPC mobilization and homing into tumors [16]. Several reports have implicated cytokines, chemokines, hypoxia-inducible 1, integrin, and MMP-9 in regulating tumor angiogenesis. Recent studies indicate that Id1 plays a role in BM-derived hematopoietic progenitor cell mobilization [17-20]. In the present study, we demonstrated that over-expression of Id1 alone can induce angiogenic processes of EPCs in ovarian cancer. Moreover, knock-down of Id1 in EPCs almost completely abolished the EPC angiogenic processes in ovarian cancer. These findings indicate a crucial role for Id1 in ovarian cancer EPCs. Id1-induced EPC angiogenesis is partially blocked by the NF-κB inhibitor (PDTC) or the PI3K inhibitor (LY294002). Activation of NF-κB by angiogenesis factors in normal cells usually increases the expression of VEGF, but not MMP-2. Interestingly, activation of NF-κB by Id1 led to the high expression of MMP-2, instead of VEGF, in EPCs from patients with ovarian cancer in the present study. This may explain why Id1 transfectants are tumorigenic.

Both Id1 and NF-κB are over-expressed in EPCs from patients with ovarian cancer, which contributes to EPC angiogenesis. NF-κB regulates MMP-2 [21], whereas Id1 strengthens this regulation via an increase of NF-κB promoter activity, which contributes to an increase of NF-κB constitutively. However, we could not exclude the possibility that Id1 reduces the tumor volume by inhibition of angiogenesis. Id1 has recently been recognized as a clinical outcome predictor in esophageal squamous carcinoma [22]. We believe that focusing on the entire Id1/NF-κB/MMP-2 signaling pathway or downstream key molecules specific for EPC angiogenesis is more relevant to clinical prognosis than an upstream molecule that has extensive effects on multiple signaling pathways. Id1 is mainly expressed in cancer cells, but is occasionally seen in epithelial basal cells and proliferating fibroblasts surrounding the tumor cells. The function of Id1 may also be offset by other HLH transcription factors, such as E-box proteins, which are involved in cellular differentiation acting against Id1 [23]. In ovarian cancer, we have observed that some Id1-positive specimens are associated with well-differentiated cancer cells. This suggests that Id1 alone does not determine the cellular fate (proliferation or differentiation). It seems that the interaction between Id1 and its antagonists (HLH transcription factors) determines the cell fate. If this is true, Id1-predominant ovarian cancer EPCs may not necessarily be poorly differentiated but surely committed to cellular angiogenesis.

## Conclusion

In summary, these data support the rationale of pharmacologic inhibition of the Id1/NF-κB/MMP-2 or Id1/PI3K/Akt pathways for ovarian cancer therapy and suggest that inhibition of Id1 or its downstream molecule MMP-2 removes the protection of ovarian cancer EPC from angiogenesis. Therefore, these EPC properties may be of significant clinical utility for ovarian cancer radiochemosensitization to improve long-term patient outcomes.

## Abbreviations

EPCs: Endothelial progenitor cells; Id1: inhibitor of DNA binding/differentiation1; MMP-2: matrix metalloproteinase-2; PDTC: Pyrrolidine dithiocarbamate; SiRNA: Small interference RNA; CDNA: Complementary DNA; SD: Standard deviation; Real-time PCR: Real-time quantitative polymerase chain reaction.

## Competing interests

The authors declare they have no competing interests.

## Authors’ contributions

SBF and SYP conceived of the study and drafted the manuscript. YJS and LJG participated in its design and helped to draft the manuscript. LCT carried out the molecular biological studies and performed the statistical analysis. YW collected the patient information. JLC helped to revise the manuscript and performed the statistical analysis. All authors read and approved the final manuscript.

## References

[B1] Kopp HG Ramos CA Rafii S Contribution of endothelial progenitors and proangiogenic hematopoietic cells to vascularization of tumor and ischemic tissue Curr Opin Hematol 2006 13 175 181 10.1097/01.moh.0000219664.26528.da 16567962 PMC2945883

[B2] Asahara T Takahashi T Masuda H VEGF contributes to postnatal neovascularization by mobilizing bone marrow derived endothelial progenitor cells Embo J 1999 18 3964 3972 10.1093/emboj/18.14.3964 10406801 PMC1171472

[B3] Urbich C Dimmeler S Endothelial progenitor cells: characterization and role in vascular biology Circ Res 2004 95 343 353 10.1161/01.RES.0000137877.89448.78 15321944

[B4] Gao D Nolan DJ Mellick AS EPCs control the angiogenic switch in mouse lung metastasis Science 2008 319 195 198 10.1126/science.1150224 18187653

[B5] Maw MK Fujimoto J Tamaya T Overexpression of inhibitor of DNA-binding (ID)-1 protein related to angiogenesis in tumor advancement of ovarian cancers BMC Cancer 2009 9 430 10.1186/1471-2407-9-430 20003244 PMC2796680

[B6] Lyden D Young AZ Zagzag D Id1 and Id3 are required for neurogenesis, angiogenesis and vascularization of tumour xenografts Nature 2007 104 1260 1265 10.1038/44334 10537105

[B7] Jankovic V Ciarrocchi A Boccuni P Id1 restrains myeloid commitment, maintaining the self-renewal capacity of hematopoietic stem cells Proc Natl Acad Sci U S A 2007 104 1260 1265 10.1073/pnas.0607894104 17227850 PMC1783103

[B8] Shaked Y Ciarrocchi A Franco M Lee CR Man S Cheung AM Hicklin DJ Chaplin D Foster FS Benezra R Kerbel RS Therapy-induced acute recruitment of circulating endothelial progenitor cells to tumors Science 2006 313 5794 1785 1787 10.1126/science.1127592 16990548

[B9] Su YJ Zheng L Wang Q Bao J Zh C Ailan L The PI3K/ Akt pathway upregulates Id1 and integrin α4 to enhance recruitment of human ovarian cancer endothelial progenitor cells BMC Cancer 2010 10 459 10.1186/1471-2407-10-459 20796276 PMC2940800

[B10] Su Y Zheng L Wang Q Li W Cai Z Xiong S Bao J Quantity and clinical relevance of circulating endothelial progenitor cells in human ovarian cancer J Exp Clin Cancer Res 2010 29 1 27 10.1186/1756-9966-29-27 20334653 PMC2857826

[B11] Tsuchiya K Kim Y Ondrey FG Lin J Characterization of a temper-ature-sensitive mouse middle ear epithelial cell line Acta Otolaryngol 2005 125 823 829 10.1080/00016480510031533 16158528

[B12] Gao DC Nolan D McDonnell K Vahdat L Benezra R Bone marrow-derived endothelial progenitor cells contribute to the angiogenic switch in tumor growth and metastatic progression Biochim Biophys Acta 2009 1796 33 40 19460418 10.1016/j.bbcan.2009.05.001 PMC3649840

[B13] Nolan DJ Ciarrocchi A Mellick AS Jaggi JS Bambino K Bone marrow-derived endothelial progenitor cells are a major determinant of nascent tumor neovascularization Genes Dev 2007 21 1546 1558 10.1101/gad.436307 17575055 PMC1891431

[B14] Peters BA Diaz LA Polyak K Contribution of bone marrow-derived endothelial cells to human tumor vasculature Nat Med 2005 11 261 262 10.1038/nm1200 15723071

[B15] Duda DG Cohen KS Kozin SV Evidence for incorporation of bone marrow-derived endothelial cells into perfused blood vessels in tumors Blood 2006 107 2774 2776 10.1182/blood-2005-08-3210 16339405 PMC1895376

[B16] Mieno S Boodhwani M Robich MP Clements RT Sodha NR Sellke FW Effects of diabetes mellitus on VEGF-induced proliferation response in bone marrow derived endothelial progenitor cells J Card Surg 2010 25 5 618 625 10.1111/j.1540-8191.2010.01086.x 20626511 PMC2958227

[B17] Spring H Schuler T Arnold B Hammerling GJ Ganss R Chemo-kines direct endothelia l progenitors int o tumor neovessels Proc Natl Acad Sci USA 2005 102 18111 18116 10.1073/pnas.0507158102 16326806 PMC1312396

[B18] Du R Lu KV Petritsch C Liu P Ganss R Passegue E Song H Vandenberg S Johnson RS Werb Z HIF1 α induces the recruitment of bone marrow–derived vascular modulatory cells to regulate tumor angiogenesis and invasion Cancer Cell 2008 13 206 220 10.1016/j.ccr.2008.01.034 18328425 PMC2643426

[B19] Li B Sharpe EE Maupin AB Teleron AA Pyle AL Carmeliet P Young PP VEGF and PlGF promote adult vasculogenesis by enhancing EPC re-cruitment and vessel formation at the site of tumor neovascularization FASEB J 2006 20 1495 1497 10.1096/fj.05-5137fje 16754748

[B20] Yang L Huang J Ren X Gorska AE Chytil A Aakre M Carbone DP Matrisian LM Richmond A Lin PC Abrogation of TGFβ signal-ing in mammary carcinomas recruits Gr-1+CD11b+myeloid cells that promote metastasis Cancer Cell 2008 3 23 35 18167337 10.1016/j.ccr.2007.12.004 PMC2245859

[B21] Dutta S Wang FQ Wu HS Mukherjee TJ Fishman DA The NF-κB pathway mediates lysophosphatidic acid (LPA)-induced VEGF signaling and cell invasion in epithelial ovarian cancer (EOC) Gynecol Oncol 2011 23 1 129 137 21782227 10.1016/j.ygyno.2011.06.006

[B22] Lin J Guan Z Wang C Feng L Zheng Y Caicedo E Bearth E Peng JR Gaffney P Ondrey FG Inhibitor of differentiation 1 contributes to head and neck squamous cell carcinoma survival via the NF-kappaB/survivin and phosphoinositide 3-kinase/Akt signaling pathways Clin Cancer Res 2010 1;16 1 77 87 20028744 10.1158/1078-0432.CCR-08-2362 PMC3321741

[B23] Mern DS Hoppe-Seyler K Hoppe-Seyler F Hasskarl J Burwinkel B Targeting Id1 and Id3 by a specific peptide aptamer induces E-box promoter activity, cell cycle arrest, and apoptosis in breast cancer cells Breast Cancer Res Treat 2010 124 3 623 633 10.1007/s10549-010-0810-6 20191379

